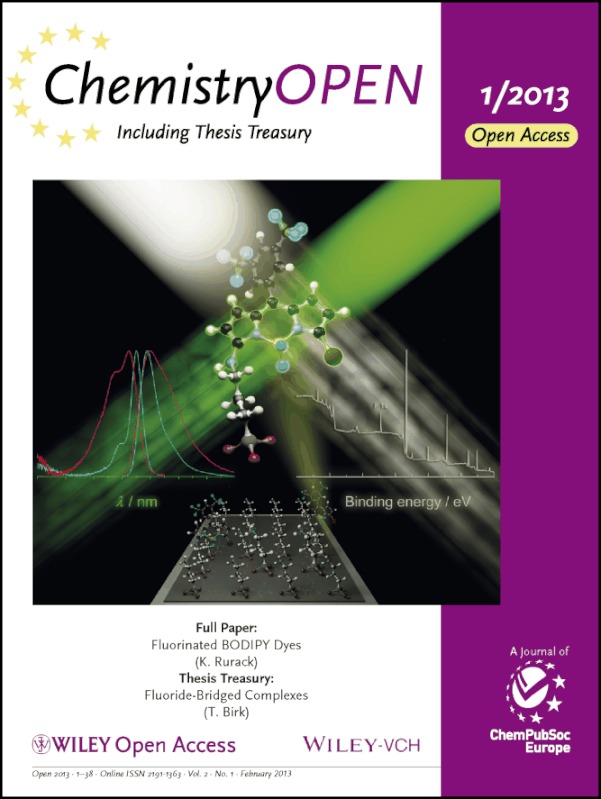# Cover Picture: Fluorinated Boron-Dipyrromethene (BODIPY) Dyes: Bright and Versatile Probes for Surface Analysis (ChemistryOpen 1/2013)

**DOI:** 10.1002/open.201390001

**Published:** 2013-02-27

**Keywords:** amino groups, dyes, fluorescence, surface analysis, X-ray photoelectron spectroscopy

## Abstract

**Cover Picture:**

Mandy Hecht, Tobias Fischer, Paul Dietrich, Werner Kraus, Ana B. Descalzo, Wolfgang E. S. Unger, and Knut Rurack*

Retrieving quantitative molecular information from macroscopic areas in a reliable manner at micrometric resolution is a challenging task, yet essential for a better understanding and control of the assembly of (bio)chemical entities on a functional surface. In their Full Paper, Knut Rurack and co-workers introduce fluorine-containing moieties to boron-dipyrromethene (BODIPY) dyes, thus making them versatile probes for dual-method surface analysis (p. 25 ff.).

**The cover picture shows** a bright fluorescent dye (green halo) containing a high number of fluorine atoms (blue) for the assessment of reactive amino groups on a glass surface by two complementary surface analytical techniques, non-destructive fluorescence scanning and quantitatively traceable X-ray photoelectron spectroscopy (XPS), both applied to the same individual samples.